# Advances in Hypofractionated Irradiation-Induced Immunosuppression of Tumor Microenvironment

**DOI:** 10.3389/fimmu.2020.612072

**Published:** 2021-01-25

**Authors:** Yuxia Wang

**Affiliations:** Department of Radiation Oncology, Peking University Third Hospital, Beijing, China

**Keywords:** hypofractionated irradiation, immunosuppression, tumor, immune microenvironment, radiotherapy

## Abstract

Hypofractionated radiotherapy is external beam irradiation delivered at higher doses in fewer fractions than conventional standard radiotherapy, which can stimulate innate and adaptive immunity to enhance the body’s immune response against cancer. The enhancement effect of hypofractionated irradiation to immune response has been widely investigated, which is considered an approach to expand the benefit of immunotherapy. Meanwhile, increasing evidence suggests that hypofractionated irradiation may induce or enhance the suppression of immune microenvironments. However, the suppressive effects of hypofractionated irradiation on immunomicroenvironment and the molecular mechanisms involved in these conditions are largely unknown. In this context, we summarized the immune mechanisms associated with hypofractionated irradiation, highlighted the advances in its immunosuppressive effect, and further discussed the potential mechanism behind this effect. In our opinion, besides its immunogenic activity, hypofractionated irradiation also triggers homeostatic immunosuppressive mechanisms that may counterbalance antitumor effects. And this may suggest that a combination with immunotherapy could possibly improve the curative potential of hypofractionated radiotherapy.

## Introduction

Radiation therapy (RT) is the mainstay of treatment in cancers, and up to 50% of cancer patients receive radiotherapy to improve local control, survival, or quality of life (1). Conventional fractionated radiotherapy usually delivers in small fractions (1.8–2.0Gy per fraction) over a number of weeks, while hypofractionated radiotherapy delivers higher dose (3–20Gy) in fewer fractions (2, 3). Emerging evidence suggests that hypofractionated radiotherapy—clinically called stereotactic body radiotherapy (SBRT) or radiosurgery (SRS)—may elicit a pronounced anti-tumor effect (4, 5). In addition to directly killing tumor cells, hypofractionated irradiation can induce tumor cells death *via* antitumor immunity (6) and vascular damage (7). There are two types of RT-induced nontargeted effects: (1) the bystander effect, which describes the additional regression of nonirradiated surrounding tumor sites after local radiation therapy (8, 9), and (2) the abscopal effect, which describes the tumor regression of distant unirradiated tumor site (10, 11). Preliminary studies suggest that radiation-induced immune responses are probably dose-dependent (12, 13). Substantial work has demonstrated that the nontargeted effects are attributed to the interaction between tumor irradiation and the host immune system (14, 15).

The tumor microenvironment (TME) is the stroma surrounding cancer cells that modulates the progression of cancer (16, 17). The TME consists of immune cells, tumor blood vessels, fibroblasts, and epithelial cells (18–21). Immune cells—such as tumor-associated macrophages (TAMs), tumor-associated neutrophils (TANs), myeloid-derived suppressor cells (MDSCs), mast cells, and natural killer (NK) cells—can produce a variety of factors (chemokines, cytokines, and enzymes) that directly or indirectly act as initiator or coordinator of the cellular immune responses to irradiation. Among all the stromal cells present in the TME, cancer-associated fibroblasts (CAFs) are one of the most abundant components of the tumor mesenchyme, which play a key role in promoting or retarding tumorigenesis in a context-dependent manner (22). In addition, radiotherapy may induce vascular damages or stimulate angiogenesis according to different regimens (23).

A large number of studies have shown that hypofractionated radiotherapy exerts a stimulating effect on the anti-tumor immune responses by inducing tumor cell death, normalizing aberrant tumor vasculature, releasing tumor associated antigens (TAAs) and inflammatory cytokines (24, 25). However, pre-clinical studies in some tumor models have suggested that radiotherapy-induced changes in the TME may induce an immunosuppressive TME, which may promote tumor invasion and spread in some situations (26, 27).

In this review, we summarized the immune mechanisms associated with hypofractionated radiotherapy, highlighted the advances in its immunosuppressive effect, and further discussed the potential mechanism behind this effect.

## Hypofractionated Irradiation Influences Immunological Responses

Higher physical or biologic dose is associated with better local control and with better survival in some cases (28–30). In clinical practice, a typical example of hypofractionated radiotherapy is stereotactic body radiation, which delivers one to five fractions of doses above 6Gy per fraction to small target volumes (31). SBRT achieved high local control in the treatment of many cancers, such as early lung cancer, brain metastases, spinal metastases, and so on. The excellent efficacy of SBRT mainly attributed to the precisely delivered high dose to tumor site and the minimized dose to adjacent normal tissue. Besides this, SBRT can induce tumor cell death and tumor size reduction in non-radiotherapy sites, named bystander effect and abscopal effect (8–11).

The radiobiological mechanisms of hypofractionated radiotherapy are largely different from that of conventional radiotherapy. The reoxygenation, repopulation, repair, and redistribution (4Rs) are important components in the response of tumor to conventional fractionated radiotherapy (32, 33), and tumor cells are killed directly through irreparable DNA double-strand breaks in the forms of mitotic catastrophe and cellular apoptosis (34). Low-dose irradiation induces DNA damage and initiate apoptosis by activation of p53-dependent mechanisms, upregulating plasma membrane death receptor, and activation of the pro-apoptotic SAPK/JNK pathway (35, 36). However, in the setting of high dose irradiation, tumor cells may be eliminated in the form of necrosis (32). Necrosis is considered an immunogenic pathway and often accompanied by the release of pro-inflammatory cytokines and damage-associated molecular patterns, which promote tumor cell killing by anti-tumor T cell response (37).

Hypofractionated radiotherapy may change the tumor cell phenotype and the tumor microenvironment. After hypofractionated irradiation, tumor cells demonstrate increased cell-surface expression of immunogenic molecules, such as adhesion molecules, death receptors, stress-induced ligands, heat shock proteins, and stimulatory molecules (such as MHC-I and CD80) (38, 39). These immunophenotype changes make human tumors more amenable to be recognized by immune system and more sensitive to T cell-mediated cytotoxicity (40). Additionally, pro-inflammatory molecules and danger signals increase in the tumor microenvironment (41–43). Immune cells, such as CD8+ T cells and dendritic cells, are activated and recruited into the tumor and play an important role in anti-cancer immunity (44).

## TME Plays a Central Role in Response to Radiotherapy

The tumor microenvironment is the internal environment which tumors depend on survival and development. TME is associated with tumor growth, progression, and metastasis (16, 17). Cancer is an extremely complex and heterogeneous disease. Cancer cells present distinct features and various mutations, which is an important clinical determinant of patient outcomes. Dynamic changes occurring in the TME cause tumor cell variants selection, which may promote the complexity of cancer heterogeneity and impact the response to different treatment strategies (45, 46).

The TME consists of tumor stroma cells, immune cells, and a variety of factors produced by these cells (chemokines, cytokines, and enzymes) (47–49). Tumor stroma cells include cancer-associated fibroblasts, epithelial cells (ECs), and mesenchymal stromal cells (MSCs). CAFs are the most abundant cells in TME and play an important role in angiogenesis and tumor growth after irradiation (22). The damage of ECs has been shown to be a major factor in the biological mechanism in response to SBRT (50, 51). Cells of the immune system include tumor-associated macrophages, tumor-associated neutrophils, myeloid-derived suppressor cells, natural killer cells (NKs), T cells, B cells, dendritic cells (DCs), and mast cells. These cells significantly differ in radiosensitivity. In general, NKs and B lymphocytes are the most radiosensitive immune cells, while DCs, CAFs, and ECs are more radioresistant cells (27). Among the immune cells, regulatory T cells (Tregs) are more radioresistant than any other kinds of T cells (such as CD8+ T cells) and B cells (27). As a result, the response of TME after irradiation varies according to the dose and fractionation schedule, which causes different outcomes in different cell types in TME.

Radiotherapy is a double-edged sword that can activate or suppress the immune response of TME under different conditions. The “hot” TME refers to a “inflamed” phenotype with highly infiltration of T cell lymphocytes (52, 53). Typical features for a hot TME include high number of effector cells (NK cells, CD8+ T cells, and Th1 cells) and functional antigen-presenting cells (APCs), and TAMs with the M1 phenotype. On the contrary, the “cold” TME refers to a “noninflamed” phenotype lack of T cell lymphocytes infiltration (52, 53). Characteristics of a cold TME include high numbers of Treg cells and MDSCs, lack of effector cells, and enrichment of immunosuppressive cytokines (47, 54). Activated NK cells and CD8+ T cells can eliminate tumor cells, while immunosuppressive TME is associated with enhanced metastasis and poor prognosis in patients (55, 56). Currently, it is not fully understood what dose and fractional radiotherapy induces the immune activated TME, and what dose and fractional radiotherapy causes the immunosuppressive TME.

## Hypofractionated Irradiation Induces Anti-Tumor Immune Responses

**Figure 1:**
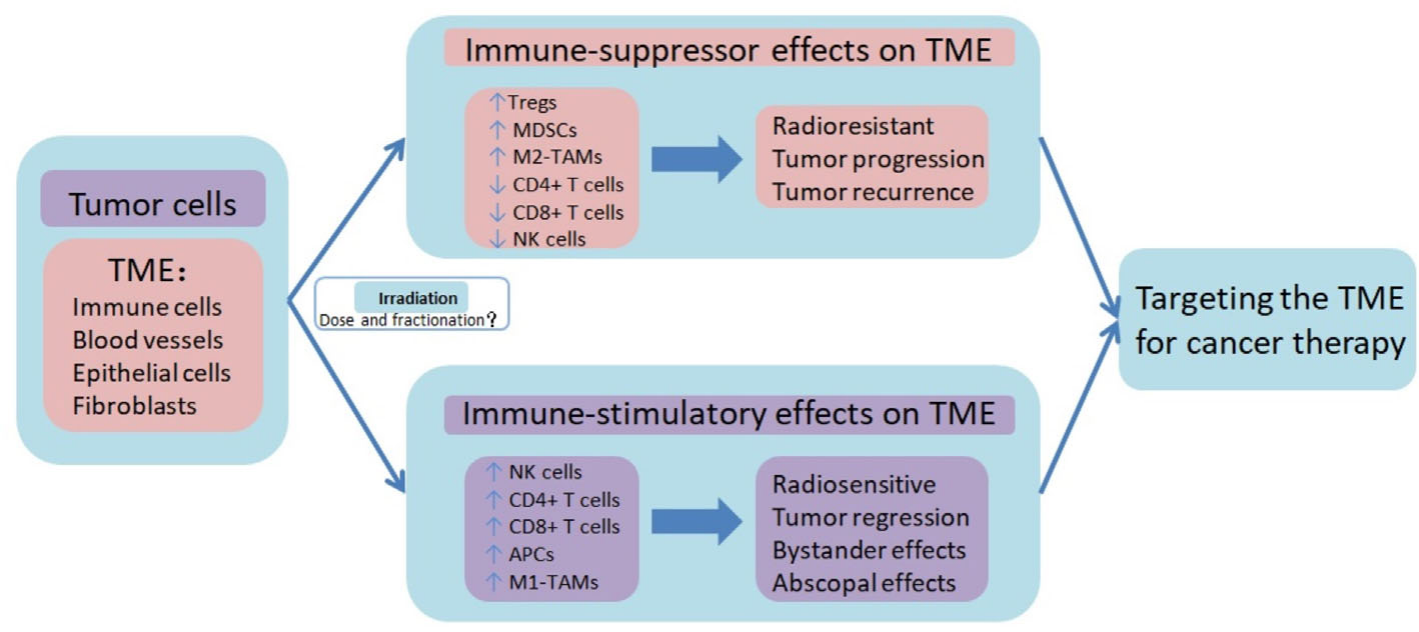
Immune-suppressor effects and immune-stimulatory effects of radiotherapy on tumor microenvironment. Radiotherapy, especially hypofractionated irradiation, contributes to the induction of anti-tumor immune responses, which promote tumor control. Besides the immune-suppressor effects, radiotherapy also induces immunosuppression of TME, resulting in tumor progression and recurrence. The high infiltration of T cell lymphocytes in TME, known as immune hot or inflamed phenotype, is characterized by high number of effector cells (NK cells, CD8+ T cells, and Th1 cells) and functional antigen-presenting cells (APCs), and TAMs with the M1 phenotype. On the contrary, the lack of T cell lymphocytes infiltration in TME, known as immune cold or noninflamed phenotype, is characterized by high numbers of Treg cells and MDSCs, lack of effector cells, and enrichment of immunosuppressive cytokines. However, it is not fully understood what dose and fractional radiotherapy induces the immune activated TME, and what dose and fractional radiotherapy causes the immunosuppressive TME. Combination therapy targeting TME may help to improve the therapeutic benefit of radiotherapy. TME tumor microenvironment, Tregs regulatory T lymphocytes, MDSCs myeloid-derived suppressor cells, M2-TAMs tumor-associated M2 macrophages, NK cells natural killer cells, APCs antigen-presenting cells, M1-TAMs tumor-associated M1 macrophages.

Emerging evidence demonstrates that hypofractionated radiotherapy can induce a pronounced anti-tumor effect ((57–62), **Figure 1**). The immunoreactive effect of radiation therapy is dose-dependent, and preclinical studies revealed that more than 8–10Gy per fraction are more effective in enhancing the anti-tumor immune response (63). In addition to the direct killing effects, hypofractionated radiotherapy can induce immunogenic death of tumor cells and orchestrate a spectrum of cellular and molecular alterations in the anti-tumor immune response (64).

After high-dose irradiation, cellular and DNA damage facilitate the generation and release of tumor-associated antigens, while necrosis or apoptosis cancer cells can generate pro-inflammatory “danger” signals and damage associated molecular patterns (DAMPs) (24, 62, 65). DAMPs and “danger” molecules stimulate dendritic cells *via* toll-like receptors (TLRs), and facilitate the uptake of TAAs and their presentation on major histocompatibility complex class 1 (MHC-1) to activate the tumor-specific cytotoxic T lymphocytes (24, 66). Dendritic cells are the major antigen-presenting cell that can process antigenic materials and present TAAs to CD8+ T cells (57, 65–67). Many preclinical studies have demonstrated that hypofractionated irradiation can increase presentation of TAAs to CD8+ T cells and enhance the antitumor T-cell-mediated immune response (68).

Irradiation also increases MHC-I expression by tumor cells, which presents TAAs to specific cytotoxic T cells, leading to the lysis of tumor cells (69). Garnett et al. (70) reported that, when irradiated by a single dose of 10–20Gy, colon and lung cell lines up-regulated the expression of MHC-I, while all of 4 prostate cancer cell lines did not. These results may suggest that the antitumor immunity response induced by hypofractionated radiotherapy differs among different cancer types.

In addition, radiation therapy exerts an immunostimulating activity by increasing NK cell cytotoxicity, facilitating the infiltration and accumulation of CD8+ T cells and tumor-associated M1 macrophages (inhibiting tumor growth), reducing the infiltration of Tregs (71), enhancing the expression of Fas and IFN-γ, and inhibiting the PD-1/PD-L1 pathway (24, 27).

Clinical evidences of the effect of radiation on TME include bystander effect and abscopal effect, in which local irradiation can induce regression in non-irradiated tumor or metastasis. Clinical reports of bystander or abscopal effects induced by radiation alone are relatively rare, and these phenomena are mainly observed in relatively high-dose radiotherapy (10). Tubin et al. (8, 9, 72, 73) conducted a series studies to explore the by stander and abscopal effects in unresectable stage IIIB/IV bulky non-small cell lung cancer (NSCLC), which were inoperable or unsuitable for radical radio-chemotherapy. They delivered 1–3 fractions each of 10–12Gy to 30% of the bulky tumor. As a result, they observed that the bystander and abscopal effects induced by partial irradiation were 95% and 45% (8), respectively. Furthermore, partial irradiation improved survival and tumor control compared to the standard of care. The researchers speculate that the induction of the bystander and abscopal effect are attribute to the irradiation to the hypoxic clonogenic cells and the sparing of peritumoral immune microenvironment and regional circulating lymphocytes. These results imply that irradiating a partial tumor may be enough to initiate immune modulation.

## Hypofractionated Irradiation Induces Immunosuppressive TME

Preclinical studies have suggested that irradiation-induced changes in tumor microenvironment may favor tumor growth, promoting tumor invasion and metastasis ((74, 75), **Figure 1**). This issue must be taken seriously, because suppression of the immune microenvironment not only leads to worse prognosis, but it may also be a legitimate therapeutic target.

Radiotherapy could eliminate radiosensitive immune cells, while radioresistant immune cells survive from it, thereby changing the proportion of immune cells in TME and causing suppression of the immune microenvironment. In the immune system, NK cells are the most radiosensitive immune cells (25, 76). On the contrary, immunosuppressive Tregs and MDSCs are more radioresistant than other population of T cells (27, 77). Kachikwu et al. (78) evaluated the impact of 0, 10, or 20Gy irradiation on Treg cells in murine model of prostate cancer. They found that Treg cells are more resistant to radiation than other lymphocytes, resulting in their preferential increase. In addition, Shi et al. (79) demonstrated that local irradiation with 10, 20, or 30Gy in cervical cancer patients significantly decreases CD8+ T cells, while having no effects on Tregs. Similarly, the MDSCs have been shown to accumulate in TME and suppress the activation of CD4+ and CD8+ T-cells (80, 81). Therefore, after certain doses and fractionated irradiation, NK cells and CD8+ T cells with anti-tumor effects are eliminated, while Treg cells and MDSCs with immunosuppressive effects are left. Changes in the types and numbers of immune cells result in the TME transformation from sensitive to resistant for response to radiotherapy (27).

High dose irradiation induces tumor vascular damage, which limits the infiltration of cytotoxic T lymphocytes into the tumor and increases the area of hypoxia (82, 83), leading to the resistance to radiotherapy (84, 85). Tumor blood vessels are more permeable and morphologically immature, and they are more sensitive to radiation (86). Vascular destruction is mainly observed at dose greater than 5 to 10Gy (27, 87), which drastically reduces the blood flow and induces hypoxia. Sonveaux et al. (88) reported that irradiation by a 6Gy dose up-regulates the expression and activity of endothelial nitric oxide synthase (eNOS). This activates the nitric oxide (NO) pathway in ECs and generates tumor angiogenesis. The process of vasculogenesis leads to the recruitment of radioresistant suppressor cells, including MDSCs, Tregs, and TAMs with the M2 phenotype (87, 89).

CAFs are the most abundant cells in the tumor stroma and play an important role in tumor angiogenesis, growth, and metastasis (90–94). CAFS are radioresistant, being able to survive at doses of up to 50Gy (95–97). CAFs can stimulate the recruitment of cells, which promotes tumor blood vessel formation and facilitates tumor recurrence (98, 99). The CAFs also secrete enzymes such as matrix metalloproteinases, which degrade the extracellular matrix, promote the migration of CAFS, and facilitate the invasion of tumor cells (90, 100). Furthermore, in vitro studies have demonstrated that a dose >10Gy to fibroblasts induces an irreversible senescent phenotype. Metabolically activated CAFs release growth factors, proteolytic enzymes, and cytokines, inducing an environment that promotes tumor growth and spread (82, 96, 101–103). However, the cancer promoting effects of senescent fibroblasts may depend on the dose and fraction of radiotherapy and vary in different tumor types (104).

Understanding the immunosuppressive effects of hypofractionated radiotherapy on the TME may help to explore new treatment strategies to block the immunosuppressive responses of radiotherapy and augment the antitumor effects (105). Previous studies suggested that both enhancing the function of tumor suppressor cells and inhibiting the function of tumor promoting cells could improve the therapeutic efficiency of radiotherapy (106–109). For example, the combination of radiotherapy and immunotherapy may prevent early exhaustion of anti-tumor immunity by boosting the activation of NK cells and cytotoxic T lymphocytes (106, 107). Moreover, inhibiting the immunosuppressive cells like Tregs, MDSCs, and TAMs can induce durable anti-tumor immunity and prevent tumor progression. A previous study by Xu et al. (108) showed that inhibition of macrophage colony-stimulating factor CSF-1 could reduce the recruitment of both TAMs and MDSCs, thereby suppressing tumor growth more effectively than irradiation alone. Furthermore, vascular-targeted agents are demonstrated to alter the tumor microenvironment to increase the radiosensitivity of tumor (110). Targeting the tumor immune microenvironment is an interesting strategy to enhance the efficacy of radiotherapy, and much remains to be investigated before realizing its potential therapeutic effects clinically.

## Discussion

Recent technological advances in external beam radiotherapy have allowed larger doses per fraction delivered to tumor, while minimizing doses to normal tissues adjacent. However, despite the increasing effectiveness of hypofractionated radiotherapy, we still can’t completely avoid tumor recurrence and progression. A large number of studies have shown that hypofractionated radiotherapy can induce immune-activated TME and improve treatment efficacy. However, increasing studies have suggested that hypofractionated radiotherapy can promote immune-suppressive TME and play a significant role in radioresistance and tumor recurrence. There is a delicate balance between TME suppression and activation triggered by hypofractionated irradiation. We still don’t know which doses and fractionation schedule activate the anti-tumor immune response and which induce the immune suppression. Moreover, different sites and types of tumors may respond differently to the same dose and fractionated irradiation. More knowledge is needed to optimize the radiotherapy strategy. Understanding the immune effects of hypofractionated radiotherapy on the TME may help to improve therapeutic benefit and explore new combination therapy strategies.

## Author Contributions

YW designed this work and wrote and revised the manuscript.

## Conflict of Interest

The author declares that the research was conducted in the absence of any commercial or financial relationships that could be construed as a potential conflict of interest.
